# J. P. Geran, D.D.S.

**Published:** 1899-07

**Authors:** 


### Obituary.

### J. P. GERAN, D.D.S.

The Dental Society of the State of New York has learned with deep regret of the death of one of its honored members, Dr. J. P. Geran, who passed away, in Brooklyn, on March 28, 1899.

Dr. Geran became a member of the Society in 1885, since which time he has been rarely absent from its meetings, serving faithfully in committees and always actively interested in all its proceedings.

Among his more intimate associates of the Second District, he was valued as a dentist of intelligence and skill, one who kept close watch upon the advances made in the dental profession, and was ready to avail himself of their assistance.

A man of good judgment, rare accomplishments, and noble qualities, he had endeared himself to a large circle of professional and personal friends, who will mourn with his family his untimely end.

